# A participatory physical and psychosocial intervention for balancing the demands and resources among industrial workers (PIPPI): study protocol of a cluster-randomized controlled trial

**DOI:** 10.1186/s12889-015-1621-9

**Published:** 2015-03-20

**Authors:** Nidhi Gupta, Christian Dyrlund Wåhlin-Jacobsen, Louise Nøhr Henriksen, Johan Simonsen Abildgaard, Karina Nielsen, Andreas Holtermann

**Affiliations:** National Research Centre for the Working Environment, Copenhagen, Denmark; Norwich Business School, University of East Anglia, Norwich, UK

**Keywords:** Randomized controlled trial, Individual, Group, Leader, Organization, Participatory approach, Need for recovery, Work ability, Sickness absence

## Abstract

**Background:**

Need for recovery and work ability are strongly associated with high employee turnover, well-being and sickness absence. However, scientific knowledge on effective interventions to improve work ability and decrease need for recovery is scarce. Thus, the present study aims to describe the background, design and protocol of a cluster randomized controlled trial evaluating the effectiveness of an intervention to reduce need for recovery and improve work ability among industrial workers.

**Methods/Design:**

A two-year cluster randomized controlled design will be utilized, in which controls will also receive the intervention in year two. More than 400 workers from three companies in Denmark will be aimed to be cluster randomized into intervention and control groups with at least 200 workers (at least 9 work teams) in each group. An organizational resources audit and subsequent action planning workshop will be carried out to map the existing resources and act upon initiatives not functioning as intended. Workshops will be conducted to train leaders and health and safety representatives in supporting and facilitating the intervention activities. Group and individual level participatory visual mapping sessions will be carried out allowing team members to discuss current physical and psychosocial work demands and resources, and develop action plans to minimize strain and if possible, optimize the resources. At all levels, the intervention will be integrated into the existing organization of work schedules. An extensive process and effect evaluation on need for recovery and work ability will be carried out via questionnaires, observations, interviews and organizational data assessed at several time points throughout the intervention period.

**Discussion:**

This study primarily aims to develop, implement and evaluate an intervention based on the abovementioned features which may improve the work environment, available resources and health of industrial workers, and hence their need for recovery and work ability.

## Background

The public finance and social welfare of western societies is challenged by demographic changes and increasing global commercial competition. Unskilled and semi-skilled workers (blue-collar workers) have an elevated risk of premature and hence costly drop-out from the labor market compared to skilled and white collar workers [1], possibly due to high physical work demands negatively influencing their health [2].

High work demands require high efforts which may deplete the resources of the workers. Over a time period, if sufficient recovery opportunities are not present, continuous depletion of resources can lead to negative effect on individual’s health and well-being and ultimately, in absence of adequate recovery, lead to negative long-term effects such as exhaustion, losses of function, and physical and mental impairment [3,4].

Recent studies demonstrate that these long-term effects are preceded by short-term effects which are indicative of acute current fatigue such as need for recovery (*a person*’*s desire to be temporarily relieved from demands in order to restore his or her resources*) ([4], p. 330)). Need for recovery is a very early stage of long-term strain process, and is noticeable in the immediate off-work situation [4]. If sufficient recovery is not achieved, a person must exert additional effort to meet the demands of job next day, and this process may start a vicious cycle leading to prolonged fatigue [4]. Empirical studies have shown that an elevated need for recovery is a predictor of high employee turnover [5], impaired well-being [6], sickness absence [5], psychosomatic [7], sleep and emotional complaints among workers [8]. Therefore, a high need for recovery after work might be a useful indication for the need to initiate preventive interventions and aim to achieve a personal psycho-physiological homeostatic balance [9].

Another scientific concept reflecting the balance between demands and resources of workers is work ability [10,11] defined as “*how good is the worker at present*, *in the near future*, *and how able is he/she to do his/her work with respect to work demands, health, and mental resources*” ([12], p. 3). A decreasing work ability has been documented to be associated with high work demands [13-15], low work resources and stress and burnout [16,17], as well as being a predictor for future sickness absence [13,18] and early retirement [13,19].

A significant proportion of the work tasks in industrial production is performed by blue-collar workers. Blue-collar workers generally experience higher fatigue and need for recovery [8,20] and increased risk of reduced or impaired work ability [21-23]. These conditions make it challenging for blue-collar workers to remain in the workforce until the age of retirement. To the best of our knowledge, few workplace interventions studies have attempted to reduce the need for recovery among blue collar workers [24-26]. Two studies have observed positive results from interventions to reduce need for recovery and increase work ability among female healthcare workers [25,26]. However, it has not been investigated if these intervention effects can be generalized to other work sectors. Similarly, many interventions have focused on improving the work ability of workers, but most studies have observed minor or no improvements [27-30]. Although the ground principle of improving work ability and need for recovery is similar (restoring the balance between demands and resources), none of the previous studies have conducted an intervention focusing on both of these concepts among blue-collar workers [24-30].

The general recommendations for conducting workplace health intervention is that success is more likely if (a) it considers multifactorial components of workers’ health [31], (b) is non-expert driven [32], (c) utilizes a structured approach for generating new action plans and subsequent implementation [33-35], (d) utilizes a participatory approach, and (e) optimally utilizes available resources in the organization to facilitate the implementation of new interventions [31]. It is furthermore important to utilize a multi-dimensional approach, focusing on organizational, psychosocial and physical (ergonomic) work environment factors, in interventions aiming to reduce the need for recovery and improve work ability, as both work ability and need for recovery concepts build on a bio-psycho-social framework [36-38]. Previous studies have predominantly used predetermined expert-developed interventions targeting need for recovery [25] and work ability [27,39]. However, expert driven interventions often result in high noncompliance and problems matching the intervention to the expectations and needs of participants [28]. Thus, tailoring of the intervention through a participatory approach has been shown to promote sustainability of the intervention, empowerment of the workers, increased commitment to the intervention, and increased credibility and implementation of the intervention at multiple levels of the organization [35,40-42]. Another key element to consider when planning an effective intervention is the optimal utilization of available resources in the organization (e.g. health and safety initiatives, management, human resources, and specialists) [31] to facilitate the intervention. The adaptation to contextual resources ensures a better fit of the intervention with the organizational culture, reduces the need for the research group or external consultants to run the intervention and likewise empowers the employees and managers who instead run the intervention, contributes to smooth implementation of the intervention and hence minimizes the financial costs for the company [35]. Finally, interventions targeting need for recovery or work ability are mostly conducted at the individual level only [24,25,27,28,39] with no or short-lasting effects [24,28]. In contrast, organizational interventions [35,43] have the potential to reduce or remove the causes of strain for entire work groups [44] and not only for vulnerable workers. Thus, organizational level interventions are generally recommended for intervening on occupational health problems [45].

To summarize, effective interventions for reducing need for recovery and improving work ability theoretically require a bio-psycho-social, participatory, and structured organizational approach, involving all levels of the organization (i.e. individual, group, leader and organizational) and optimally using the onsite resources.

Therefore, the aim of our study is to evaluate the effectiveness of an intervention based on the aforementioned features on need for recovery and work ability among industrial workers. The objective of this paper is to present the background, design, protocol and evaluation of the “Participatory Physical and Psychosocial Intervention for Balancing the Demands and Resources among Industrial Workers” (PIPPI) intervention. The primary hypothesis of PIPPI is that the intervention will decrease the need for recovery and improve the work ability of the workers.

## Methods

The CONSORT statement is utilized to describe the design and protocol of this study [46].

### Study design

**Figure 1:**
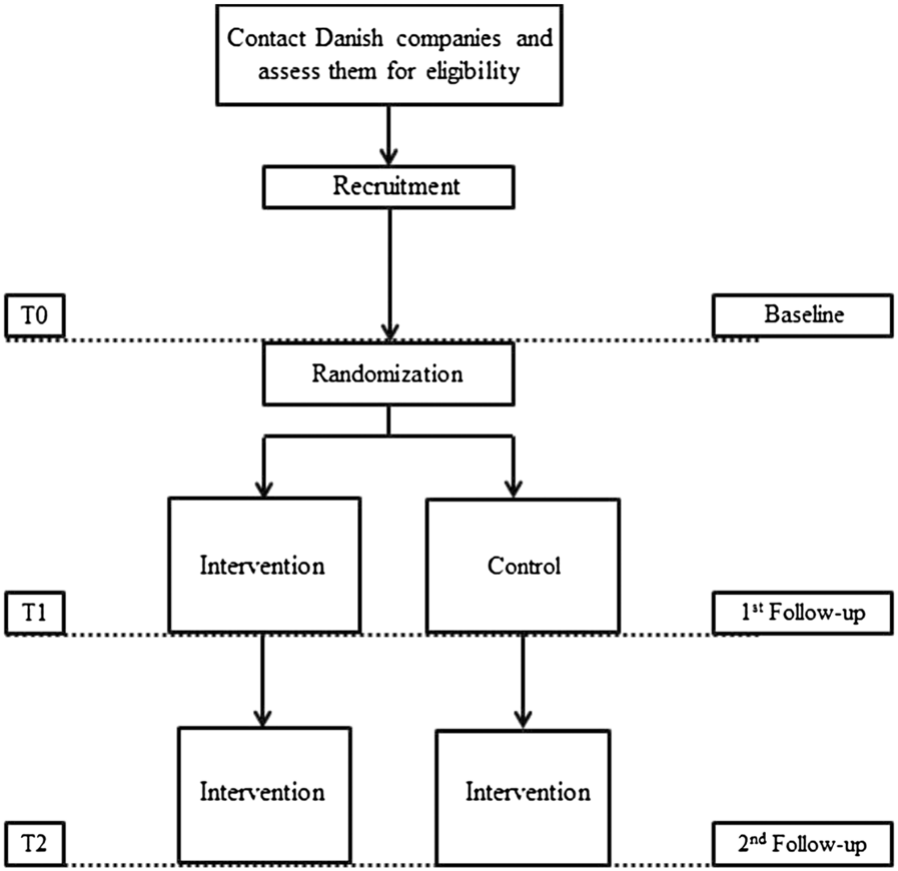
**Design of the Participatory Physical and Psychosocial intervention for Balancing the Demands and Resources** **(PIPPI) cluster randomized controlled study.** T0 = time at baseline, T1 = Time at first follow-up (12 months after baseline), T2 = Time at second follow-up (12 months after first follow-up).

The study is a two-year cluster-randomized trial in which controls also receive the intervention in year two (Figure 1). The abovementioned primary hypothesis will be tested in the first year of intervention, while the sustainability of the intervention will be analyzed in the second year of the intervention.

This trial has been registered in the Danish Data Protection Agency register (Journal number: 2013-54-0329) and in the International Standard Randomized Controlled Trial Register (ISRCTN76842602, date assigned: 10 July 213)). Moreover, this study has been approved by the Ethical Committee for the regional capital in Denmark (Journal number: H-2-2013-FSP13) and will be conducted in accordance with the Helsinki declaration [47].

### Study population

This study aims to recruit larger industrial workplaces in Denmark employing workers organized in teams mainly carrying out manufacturing work.

The inclusion criteria at the *workplace level* is that the workplace should (a) have at least 100 employees mainly involved in manual labor work, (b) be team based, (c) have a fairly cooperative relation between different levels of organization, (d) be willing to implement the PIPPI activities and (e) reflect the geographical and organizational distribution of Danish industrial production companies. Inclusion criteria at the *team level* are that the employees are organized in teams of formal work groups. Inclusion criteria at the *individual level* are that the participants should (a) work for ≥20 hours/week and (b) give consent to participate in the scientific evaluation of the study. PIPPI is an organizational-level intervention and all workers in the intervention group will participate in intervention activities. Thus, there are no individual exclusion criteria for participating in the intervention activities. However, only the workers who satisfy the individual level criteria will be included in the effect evaluation of this study.

### Recruitment

Recruitment of the companies will be done by collaborating with worker and workplace/trade unions and industrial associations. Based on the previously mentioned inclusion criteria at workplace level, the unions will be asked to provide a list of potential participating companies. The research group will contact the management of the suggested companies via email and phone, and describe the content and requirements for participating in the study. If the management shows interest in participating in the study, a meeting will be conducted for the management and worker representatives of the workplace and research the possibility for participation in the study. Once the collaboration is confirmed, details about the team level recruitment criteria, organization of the project, communication plan, intervention content, intervention implementation at all levels and establishment of local project steering group consisting of management and worker representatives, human resources employees and researchers, and their responsibilities will be explained and settled.

An information meeting will be arranged for workers where the signed consent form will be obtained from workers who wish to participate in the study. Workers will also receive information about the intervention via information meeting videos uploaded at the workplace’s homepage, via distribution of information leaflets and via posters displayed on accessible locations. The interested workers will sign informed consent forms which will be collected with the support from team leaders and key contact person at the workplace.

### Randomization

The working teams will be randomized to either the intervention group or control group utilizing cluster level randomization. A randomized block design will be utilized to prevent contamination between participating adjoining team. Thus, the working teams will be structured in blocks based on their geographical proximity and common leaders. These blocks will then be randomized into either control or intervention groups using a computer generated randomization schedule. In the second phase of the intervention, the control group will also receive the intervention.

### Intervention development and planning

Two steering groups will be established at each company. One is a strategic steering group consisting of a managerial group (often the managerial team) responsible for long term adaptation and evaluation of the usefulness of PIPPI, and the other is a project steering group responsible for monitoring and supporting the day to day activities of PIPPI. The steering group will manage the overall intervention process and ensure intervention progress at all levels of the organization throughout the project period. The steering group will (a) support data collection for effect and process evaluation, (b) discuss and monitor the screening process and developed action plans, (c) facilitate participation in intervention activities, (d) follow-up on progress, (e) reflect on the factors which will facilitate or hinder the progress of intervention, (f) discuss the evaluation of organizational resources and the corresponding action plan and (g) develop a communication strategy to ensure distribution of proper and timely information to the participating workers, managers and support systems.

The individual (I), group (G), leader (L), and organizational (O) levels of the workplace, referred to as IGLO [48], will be actively involved in the intervention activities. It signifies an approach that seeks to coordinate and integrate intervention activities at various levels of the organization. The aim of this approach is to achieve a thorough implementation and support throughout the organization. The specific intervention activities are listed below according to the level of the organization involved.

#### Group level

At the work team level, workshops are conducted to map the work environment, create action plans, and monitor the implementation of these action plans [48]. The three designated workshops are carried out by the work team together with a consultant or person from the research group using a participatory approach. A member of the research group will be present at all workshops to observe the process, and all workshops will be documented with audio recordings.

The first workshop is a collaborative screening process labelled a “Visual Mapping Workshop” (VMW) based on cognitive mapping interview techniques [49]. The VMW will focus on producing a visualization of the factors affecting the team member’s ability to work, i.e. positive and negatives aspects of work (based on the job demands-resources model [50]. Furthermore the workers are given time to construct an individual map of the factors affecting their work ability, both positively and negatively.

At the subsequent Action Planning Workshop (APW), the participants will revisit the visual map developed at VMW and use it to create action plans aiming to restore or improve the balance between demands and resources of the workers. The consultant will support the participants in the APW in making action plans that list the goals and responsibilities of individual team members and the time frame of the developed action plans. Likewise, the cost-effectiveness of all action plans are to be discussed and evaluated in a structured process focusing on their effectiveness (how many workers will be affected), and scale of effect (small-large effect) with respect to productivity, wellbeing and product quality, and their expected costs (e.g. working hours used, cost of modifying the production, or prize of new equipment). Subsequently, the developed action plans are prioritized based on their evaluated cost-effectiveness.

It will be emphasized that, in cases where there is no need for approval from senior management level or additional funding to the developed action plans, they should be implemented in the context of the work team. However, when the developed action plans involve large-scale organizational changes or additional funding, information of the action plan will be carried back to the steering group and discussed for being implemented.

The last workshop is a follow-up workshop (FUW), where the implementation of the previously developed action plans is assessed. There are clear recommendations in the occupational health psychology literature to support the implementation process, as studies have shown that the implementation conducted by the workers and line managers is often a weak point in organizational interventions [51,52]. To address this weakness, the FUW provides an opportunity for the line manager and employees to discuss the action plans with a process consultant provided by the research team.

Between the APW and FUW, sufficient time will be provided for employees and line managers to implement the proposed action plans, evaluate them, and if necessary modify or develop new action plans.

#### Leader level

At the leader level (where PIPPI will include health and safety representatives as experts on work environment issues), a one-day Ambassador Workshop (AW) will be conducted at each participating company for line managers, union and health and safety representatives. The objective of the workshops is to provide them the sufficient information and skills to take on the role of ambassador for the intervention and help the workers understand and gain access to information about the intervention. Participants in the workshops are trained to facilitate the implementation of developed action plans. Furthermore, the line managers will be trained to take a central role in the participatory process at the team and individual level (described below) as they are to offer individual work ability talks with each employee, as a supplement to the activities on the group level.

The AW includes (a) information about central guiding elements of the intervention such as participation, the demands-resources model, and the IGLO concept, (b) discussions about the expectations to the intervention as well as workers’ readiness for change, and (c) discussions regarding their roles supporting the intervention process. The AW will ensure that the line managers, union and health and safety representatives understand the overall aim of the intervention, the background for the PIPPI project, and requirements for a participatory approach. The discussions will facilitate development of common goals for the intervention, individual goals regarding their individual leadership capabilities and functioning of their work teams.

The AW will be supplemented with Learning Workshops (LWs) for the same group of line managers, union and health and safety representatives. The aim of the LWs is to discuss how they can support the implementation of action plans, and enable exchange of experiences between the participants about the progress of the intervention and the challenges faced along the way. The LW will be conducted after the first year of the intervention activities (described below).

#### Individual level

On a voluntary basis, an individual visual mapping sessions between interested employees and their line managers will be arranged. In these sessions, the principles of the first two workshops (VMW and APW) will be used at an individual level. The line manager will ask workers to individually identify key resources and demands in their work environment currently limiting or enhancing their work ability. This will provide the worker and the team leader with an overview of both the demands that could be modified and the resources that could be promoted in order to improve the work ability and reduce need for recovery for the worker. The session will result in the worker and team leader together developing future new action plans to address the specific elements leading to higher need for recovery and limiting the work ability of the individual worker if the workers wished it.

#### Organizational level

The intervention seeks to use existing organizational resources and facilities as well as the existing health and safety initiatives at the workplaces to facilitate various workshops and implementation of the intervention. An audit will be carried out aiming to map the organizational resources related to worker’s health and the work environment, as well as their current functioning [48]. Relevant workers in the organizational support systems as well as a representative sample of health and safety worker representatives will be interviewed and the formal and informal resources will be mapped, explained and evaluated. Utilizing a snowball sampling approach, each representative will also be asked for additional functions relevant for inclusion. Further interviews will be conducted with employees and managers regarding the use and function of these resources. Furthermore, existing written material related to the different functions and resources will also be collected from the workplace. A report will then be produced, summarizing the aforementioned resources as well as the perceptions about their effectiveness throughout the organization. The report will then be discussed in meetings with the project group and in the steering group. Subsequently, the additional action plans will be produced for the intervention group based on the results of the audit of organizational resources (ORA).

### Implementation of the intervention

For implementation of the action plans, the workplace will be provided with visual boards based on lean manufacturing also known as kaizen boards [53,54]. The boards can be used by the work teams as aids for discussing the progress of the action plans. In case the workplace does not already use similar systems, the workers will be instructed in how to use them. The visual boards contain areas for listing action plans currently being implemented by the team, new action plans, and details about the roles and responsibilities of individual employees or managers in implementing respective action plan. The board also allows easy charting of the progress of activities and supporting the discussion of action plans at regular team meetings. Kaizen boards follow a PDCA cycle (i.e. plan, do, check and act) in which the workers propose courses of action, implement them, evaluate their effectiveness and make adjustments to increase the efficacy of the courses of action [53]. The progress of the intervention will be discussed in the team meetings, commonly occurring at least every month, and on bi-weekly workers’ existing meetings where the teams are to briefly discuss the progress of the action plans.

The visual mapping developed at the team level will be analyzed and an overview of commonly mentioned demands and resources will be provided to the steering committee. Thus, if the workers indicate problems with the current systems and initiatives that were not identified during the ORA process, these problems can still be acted upon at a higher level than just the team, which are often unable to make decisions affecting overall workplace policies.

Another strategy of implementation is that the aid of an experienced ergonomic consultant will be available to the teams. Three hours of consultant assistance per work team will be allotted as part of the intervention, but the workplace can also bring in their own internal consultants or relevant persons of competence (e.g. production planners, machine experts) on a case-by-case basis if deemed relevant.

### Evaluation of the intervention

The evaluation framework of the intervention will comprise a comprehensive quantitative effect evaluation as well as a thorough process evaluation drawing on both quantitative and qualitative sources, i.e. a mixed methods approach.

#### Effect evaluation

**Figure 2:**
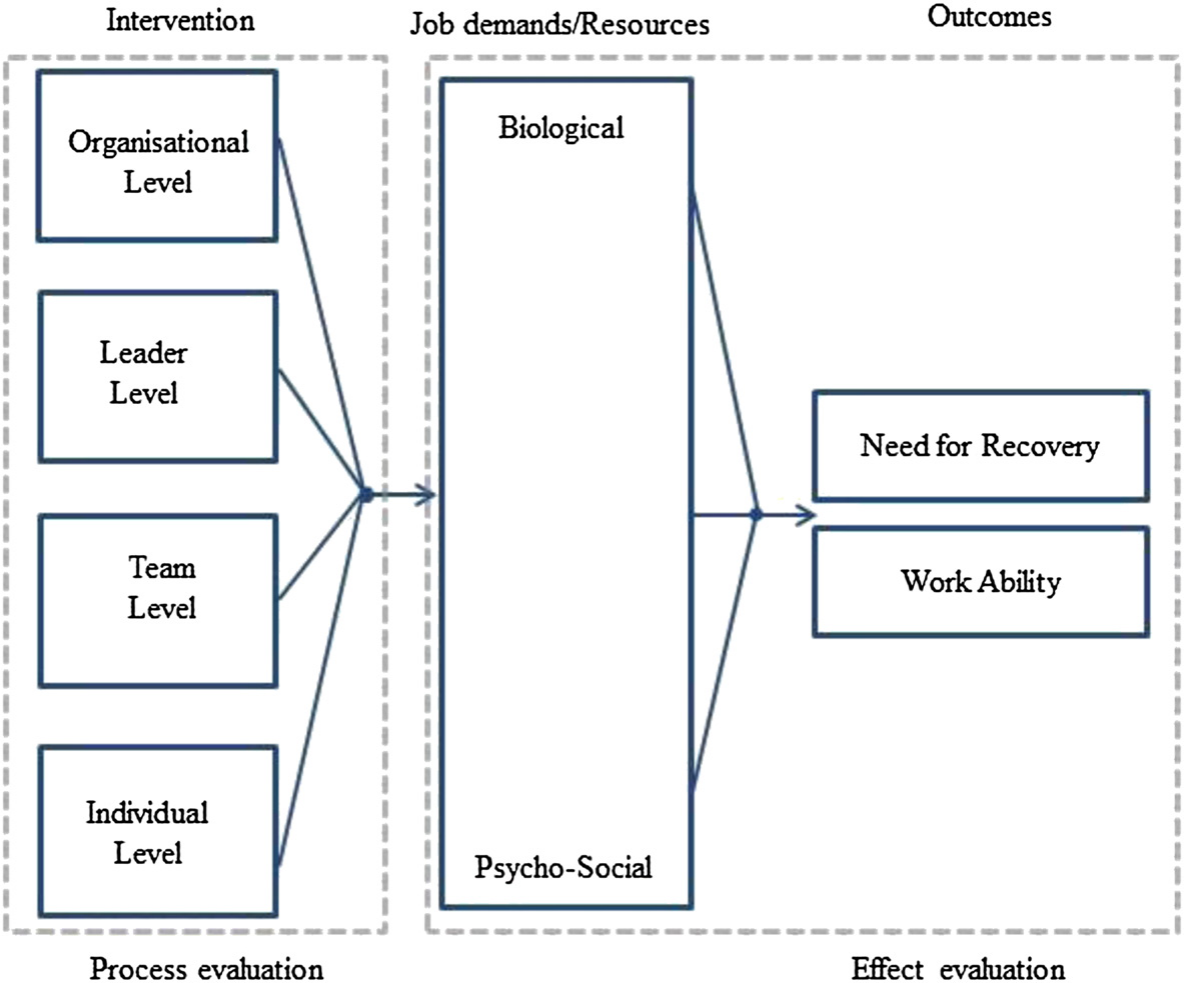
**Conceptual model for improving the need for recovery and work ability of workers.** Each strand of the intervention either generates action plans to improve the balance between work demands and resources, or assists the implementation of developed action plans. The improved balance between work demands and resources from implementation of the action plans is considered to improve the primary outcomes of this study.

The primary outcome of this study is the need for recovery and work ability [23,55]. Secondary outcomes are work demands and resources [23,56], and productivity [23]. The conceptual model to improve upon these outcomes is illustrated in Figure 2.

#### Process evaluation

The quantitative-qualitative process evaluation framework at IGLO levels utilized in this study is inspired by Nielsen and Abildgaard [51]. The objective of the process evaluation is to identify the working mechanism of change based on four categories: *Organizational actors* or drivers of change which includes all key stakeholders (employees and management supporting intervention) who may influence the intervention process and therefore the intervention outcomes, *mental models* which includes cognitive appraisal of the organization, working conditions and the intervention program and its activities [57] that can help explain the behaviors of the organizational actors, *contextual* factors which hinders or facilitates intervention activities, and *interventions design and process*. Data will be collected at different levels of the organization to address specific issues and elements related to initiation of the intervention, development and implementation of intervention activities, identification of drivers of change, participatory approach involving the employees, senior management support, role of middle management and consultants, information and communication strategy of the intervention, screening, action planning, omnibus, discrete and contextual factors and workers’ mental models of the intervention and their work situation. Case study analyses [58,59] of the working mechanisms of the intervention are planned to be conducted based on a narrative approach to study organizational processes [60,61]. Furthermore, process data are planned to be used descriptively to document the degree of implementation in a mixed methods design. This allows us to evaluate if the intervention has been conducted as planned, and to investigate associations between process data and effects on the primary and secondary outcomes. The extensive process data collection using multiple data sources is preformed to ensure that potential changes in the companies or deviations from the planned intervention are documented thoroughly. It will hence be possible on the basis of a thoroughly pre-planned and standardized qualitative and quantitative process evaluation to conduct analyses on these elements.

### Data collection

**Figure 3:**
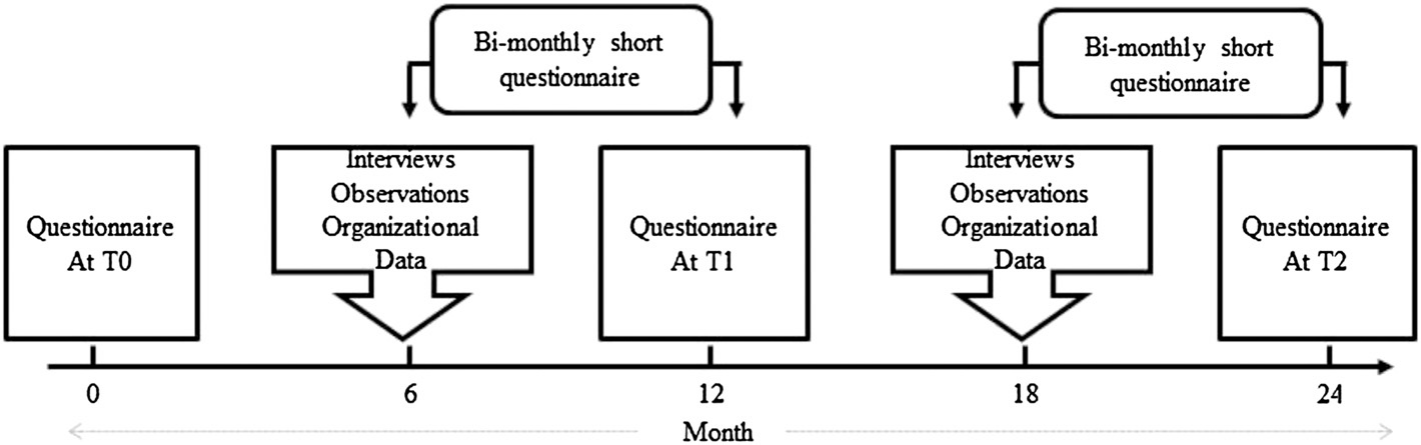
**Data collection for effect and process evaluation.** The data for process evaluation will be collected via (a) observation and audio recordings made during all main intervention activities, (b) questionnaires distributed to all team leaders, (c) various organizational documents such as evaluation forms from activities and meeting minutes collected during the course of intervention, and (d) interviews conducted at different levels of organization (example. health and safety representatives, human resources personnel, steering group members, and line managers and workers from both the intervention and control group). For collecting information for both effect and process evaluation, a main questionnaire at T0, T1 and T2 will be used.

The data from all IGLO levels will be collected via four methods: questionnaires, interviews, observations, and organizational records (Figure 3).

The main questionnaire will consist of questions covering the primary and secondary outcomes, and process evaluation. Need for recovery will be determined using 9 items (eg. “how often do you have difficulties to relax after a workday”). The five responses categories are: 0 = never, 1 = rarely, 2 = some of the time, 3 = most of the time, and 4 = always. The need for recovery index will be composed based on the mean of all items. Work ability will be determined via the single work ability item (i.e. rating of the current work ability compared with the lifetime best) with response ranging from 0 (“unable to work”) to 10 (“work ability at its best) [62]. Besides the main questionnaire, need for recovery and work ability items are also included in a short email questionnaire which will be responded to by the participants two and four months prior to the follow-up main questionnaire. Process evaluation questionnaire items will be included in the follow-up questionnaire and will be based on scales and items from Randall and colleagues [63] and Nielsen and colleagues [64], adapted to fit the intervention context of industrial production (sample items include “my immediate manager was positive about the implementation of PIPPI” and “the project had led to sustainable changes in their workplace”). Data for process evaluation will be collected via audio recording of all group level workshops supplemented with observations of the workshop by a member of the research team adhering to a standardized set of instructions focusing on non-verbal aspects (i.e. elements that are not captured on the audio recording). Organizational data (action plans, meeting minutes etc.) are collected and semi structured interviews [65] are conducted in all teams using a standardized interview guide. Interviews will be conducted shortly prior to the effect evaluation questionnaire.

The work demands such as duration of lifting and carrying, pushing and pulling, physical exertion at work, duration spent in arm at or above shoulder, back bending forward and the resources such as physical fitness and strength will be measured using questions from a Danish national survey questionnaire [66]. Productivity at work will be measured using a validated single item ‘how would you rate your productivity at work’ in the last month, with responses ranging from 0 (worst) to 10 (best productivity). Psychosocial demands and resources will be measured using the WHO5 well-being index (5 items, sample item. during the last 4 weeks I have felt calm and relaxed) and the mental health subscale from the SF-36 questionnaire (3 items, sample item. during the last 4 weeks have you been very nervous) [67] both using 6 response categories ranging from “all the time” to “at no time”*.*

### Statistics

The effect of the intervention on the primary and secondary outcomes will be performed using a multi-level mixed model or generalized linear mixed models (GLMM) based on comparison between the intervention and the control group from baseline (T0) to 12 months after baseline (T1). Four levels will be included in the mixed model: time (measurement time points), worker, working team and enterprise. The multi-level analyses will concurrently take into account the clustering of observations of workers within the working team, as well as repeated measurements within each worker. Sensitivity analyses will be conducted under different assumptions about missing data [68].

### Sample size calculation

The sample size was calculated based on the standardized effect size, a standardized difference between intervention and control groups. On the one hand, the PIPPI intervention is extensive, targeting a range of challenges in the work environment at various levels of the organization. On the other hand, the intervention is not equally intensive for every employee – the intensity depends on a number of circumstances, e.g. the number of action plans designed and completed in the work group, or whether the employee participates in the individual visual mapping session with his or her line manager. Thus on average, we assumed a medium effect size, corresponding to a standardized difference score of 0.50 [69].

Because we are not aware of any previous similar intervention study among manufacturing workers, we assume an intra-class correlation of .05 based on a previous cluster randomized controlled study on prevention of low back pain and its consequences among nurses’s aides in elderly care in Denmark [70]. With level of significance (α) of .05, a statistical power (1-β) of 0.9, intra-class correlation of .05, and a design effect of 1.54 (considering average cluster size of 22 workers), 200 (9 teams) workers in each group will be required.

Generally, workplace intervention studies have a high dropout rate. Also, there is a risk of organizational changes happening at workplace which can lead to drop-out of entire cluster from the evaluation. Therefore, we are aiming to randomize more than 400 workers (more than 18 teams) in both groups.

## Discussion

This paper has presented the design and protocol of a cluster randomized controlled trial aiming to reduce the need for recovery and improve the work ability of industrial workers via balancing their work demands and resources.

### Impact of results

The hypothesis of PIPPI is that, if effective, the intervention will improve work resources, modify work demands and consequently reduce need for recovery and improve the work ability of industrial workers. This study will add to the existing scientific literature about developing effective interventions targeting all levels of the organization for improving need for recovery and work ability.

This intervention will aim to encourage the workplace to gradually take responsibility for the different parts of the intervention, so that organizational learning is achieved, and the workplace attains the sufficient competence for maintaining the PIPPI intervention after the first year of the intervention. Therefore the program theory is twofold: primarily, implementation of the developed action plans for improving resources and modifying work demands are thought to positively influence work ability and need for recovery. Secondly, the empowerment of employees gained from the participatory approach combined with the learning achieved by the line managers, union and health and safety representatives will improve the capabilities of the company to maintain the intervention for a prolonged period of time. Thus the short and long term goals of PIPPI is to continuously improve on the ergonomic and psychosocial working conditions, reducing the strenuousness of working in industrial production, improving the work ability and decreasing the need for recovery. There are many interventions available seeking to restore balance between work demands and resources, but most of these require extensive resources which may not be available to the workplace. For example, de Boer and colleagues [28] administered individual counseling and an educational program run by occupational physicians hired from an independent labor organization. After two years, the intervention effect was not significant. The authors stated that follow up sessions of the intervention may be necessary to sustain the effect of the intervention. However, to sustain such an intervention will require an effort that is likely not to be cost-efficient. Our intervention will address this limitation by: (a) adapting the intervention activities to the structures and available resources at the workplace (b) training internal consultants and line managers to effectively conduct the PIPPI activities themselves in the future and (c) providing knowledge on improving the routines of managing the health and safety at work. These features will potentially lead to more sustainable implementation of the intervention at the workplaces.

The intervention activities may also provide knowledge on how to improve the balance between work demands and resources of blue-collar workers in other sectors. Although this intervention is conducted in the industrial sector, it is based on general organizational intervention principles which are also applicable in other sectors. This study will provide an organizational intervention framework which could be utilized, with some modifications in other occupations and could potentially be developed for skilled and white collar workers as well.

### Strengths

The present study has several strengths, One strength is that it builds on several recommendations for conducting effective work environment and health promotion interventions at workplaces, especially regarding involvement and support throughout the process [51,71,72]. The proposed intervention fulfills several criteria of effectiveness of workplace interventions to prevent and manage common health problems suggested by Hill and colleagues [73]. These criteria are that an intervention (a) ought to include some form of partnership between employer/employees and consultants, (b) should be conducted at the workplace, (c) take into account workers’ attitudes and beliefs, (d) be comprehensive in nature, addressing both individual and organizational factors, and (e) aim to improve communication, cooperation and common goals between employers, employees, and occupation health providers. These criteria will be fulfilled in the present intervention by (a) including employers, employees and consultants at each stage of the intervention from planning to evaluation, (b) utilizing an organizational intervention and considering the attitude, wishes, belief, and priorities of employees in a participatory approach while planning the intervention, (c) developing a multi-dimensional comprehensive intervention considering different factors at IGLO levels, and (d) developing an effective communication plan to increase cooperation and shared goals between employers and employees. Another strength of this study is the cluster-randomized controlled evaluation design which is by many considered the gold standard for evaluating these types of interventions. Moreover, the combined effect and process evaluation framework will provide information on why, how, in which circumstances and for whom the intervention worked [74]. The comprehensiveness of the process evaluation will make it possible to conduct detailed analyses of the working mechanisms of PIPPI.

### Limitations and risks

The first limitation of this intervention is that the action plans generated through the workshops and participatory approach may differentially target combinations of work demands and resources among the working teams. However, this aspect of the PIPPI intervention also acts as a strength of the study as this tailors the intervention activities to the specific needs, resources, wishes and barriers of the participating workers. The heterogeneity of the intervention content and implementation may therefore be necessary in order to obtain positive effects. Finally, the limitation of a participatory approach is that some action plans may focus on areas that are perceived by employees as highly relevant, but are unrelated to need for recovery and work ability. Though this is unlikely for the majority of action plans, we collect information on all actions developed to monitor the content and targets of the activities.

Another limitation is that the control group may start activities to improve work ability and need for recovery themselves or even seek some of the intervention activities on their own or inadvertently receive some aspects of the intervention (e.g. because of intervention activities initiated from a higher organizational level). This limitation will be addressed by using a cluster-randomization which will reduce the contamination between the groups by ensuring that the groups are not sharing the same geographical area and managers. Additionally, the steering group will also be informed about this issue to ensure that the actions developed by the intervention group are not implemented among the controls in the first follow-up period.

A potential risk factor for this trial is the senior management’s interest and motivation in the project which may diminish if the intervention fails to make progress according to their expectations. The role of senior management is vital for successfully planning, implementing and evaluating the intervention [75]. To address this risk, we will utilize different strategies to ensure managers readiness for change and motivation, such as involving them in the steering committee, and ensuring support for the intervention from them by discussing the intervention content with them.

Another potential risk for this trial is the failure of line and middle managers to fulfill their intended roles. These managers are responsible for the daily progress, communication and implementation of intervention activities. Studies have observed both passive [76] and active [77] resistance by these managers as driver of change in organizational change processes, influencing the outcome negatively [52,78]. This risk factor will be addressed in the present study by thoroughly informing the line and middle managers of the actual behaviors which they must perform in each phase of the intervention. Also, interviews exploring the mental models of the line and middle managers regarding the intervention will be carried out enabling us to evaluate what role these managers actually saw themselves as fulfilling and how they supported the intervention.
